# High mesothelin expression is associated with low cytotoxic T cell infiltration in pancreatic cancer

**DOI:** 10.3389/fimmu.2025.1651687

**Published:** 2025-10-08

**Authors:** Oliver Liang, Amy L. White, Timothy Fielder, Joo-Shik Shin, Sharon M. Sagnella, Ulf Schmitz, Dannel Yeo

**Affiliations:** ^1^ Precision Oncology Laboratory, Cancer Innovations, Centenary Institute, Sydney, NSW, Australia; ^2^ School of Medical Sciences, Faculty of Medicine and Health, The University of Sydney, Sydney, NSW, Australia; ^3^ Department of Tissue Pathology and Diagnostic Oncology, Royal Prince Alfred Hospital, NSW Health Pathology, Sydney, NSW, Australia; ^4^ Central Clinical School, Faculty of Medicine and Health, The University of Sydney, Sydney, NSW, Australia; ^5^ Cell and Molecular Therapies, Royal Prince Alfred Hospital, Sydney Local Health District, Sydney, NSW, Australia; ^6^ Computational Biomedicine Lab, College of Science and Engineering, James Cook University, Townsville, QLD, Australia

**Keywords:** biomarker, microenvironment, immunosuppression, immunotherapy, precision medicine

## Abstract

**Objectives:**

Mesothelin (MSLN) is a cell-surface glycoprotein overexpressed in the majority of pancreatic ductal adenocarcinoma (PDAC) cases and represents a promising immunotherapeutic target. Despite studies and clinical trials investigating MSLN-targeted immunotherapies, its biological role in PDAC carcinogenesis and influence on the tumor microenvironment remain poorly characterized. This study aims to investigate MSLN expression patterns in PDAC and assess their relationship to clinical outcomes and the immune microenvironment.

**Methods:**

MSLN expression in 74 PDAC patients was evaluated by immunohistochemistry staining on a tissue microarray and correlated with clinicopathological features and survival outcomes. Complementary analyses of publicly available transcriptomic datasets (bulk RNA-seq and single-cell RNA-seq) were performed to characterize associations between MSLN expression and the tumor immune microenvironment with immunohistochemical validation.

**Results:**

High MSLN expression (H-score ≥ 62) was associated with improved relapse-free survival (p = 0.021) and with increased patient age (p = 0.036). Transcriptomic analyses revealed high MSLN expression was associated with an immunosuppressive microenvironment characterized by reduced immune reactivity and diminished cytotoxic T cell infiltration. Immunohistochemical validation confirmed a trend toward decreased stromal cytotoxic T cell abundance with increasing MSLN expression.

**Conclusion:**

This study revealed an inverse relationship between MSLN expression and cytotoxic T cell infiltration in PDAC, despite a trend toward improved relapse-free survival in MSLN-high tumors. These findings have important implications for MSLN-targeted immunotherapies and suggest that addressing the immunosuppressive microenvironment may be necessary to optimize their current responses in PDAC.

## 1 Introduction

Pancreatic ductal adenocarcinoma (PDAC), accounting for more than 90% of all pancreatic malignancies, is an aggressive and lethal cancer (1). PDAC has a poor prognosis with a rising incidence rate and a high mortality rate (five-year survival of less than 10%) (2, 3). The majority of PDAC cases arise from microscopic dysplastic lesions known as pancreatic intraepithelial neoplasms (PanINs) (4), although other cystic precursor lesions, such as intraductal papillary mucinous neoplasms (IPMNs) and mucinous cystic neoplasms (MCNs), can also become malignant (5). Diagnosis occurs late in the majority of PDAC patients due to both the absence of specific clinical symptoms during early disease and the inherent challenges in imaging and detecting early-stage pancreatic tumors (6, 7). Although surgical resection is the only potentially curative treatment, most patients are diagnosed with locally advanced or metastatic disease and as such, are not eligible for resection. Standard systemic chemotherapy and radiotherapy have shown limited efficacy to date, highlighting the need for more effective therapies (8, 9).

MSLN is a glycosylphosphatidylinositol (GPI)-anchored glycoprotein that is overexpressed in certain solid tumors including PDAC, with minimal expression in normal tissues. Anti-MSLN immunotherapies, such as antibody-based therapeutics (10–12), immunotoxins (13), antibody-drug conjugates (14), and chimeric antigen receptor (CAR) T cells (15–17), have been evaluated in clinical trials. Despite promising preclinical results (18–20), clinical response to anti-MSLN immunotherapies remains modest (21, 22). Efforts are ongoing to better understand the underlying cause of treatment failures and to improve the effectiveness of the therapy.

The clinical significance of MSLN expression has been studied in PDAC as well as other cancer types including colorectal cancer (23, 24), ovarian cancer (25, 26), breast cancer (27, 28), gastric cancer (29, 30), lung cancer (31, 32), and mesothelioma (33, 34). However, there are conflicting results on the prognostic potential of MSLN due in part to differences in cohorts and methodologies used. Cohorts from the United States and Japan have reported an unfavorable association with tumor pathology and/or survival outcomes based on MSLN transcript (35, 36) and protein (37, 38) levels. Although no survival analysis was undertaken, no association was found between MSLN expression in PDAC tissues and clinicopathological factors (age, sex, disease stage, and tumor differentiation) in one cohort from China (39). No studies, to date, have performed immunohistochemical evaluation of MSLN in an Australian PDAC cohort.

In addition to its therapeutic and clinical significance, the biological importance of MSLN remains poorly understood. Under normal physiological conditions, MSLN is lowly expressed in mesothelial cells of the pleural, peritoneal, and pericardial lining (40). The physiological function of MSLN remains elusive, as MSLN knockout mice do not display abnormalities in survival, development or reproduction (41). In cancer, MSLN is involved in various pathways that promote tumorigenesis. PDAC cells overexpressing MSLN promote proliferation by activation of STAT3 (42). MSLN signals through the PI3K/Akt pathway to increase autocrine IL-6 production and protect PDAC cells from TNF-alpha induced apoptosis (43–45). MSLN also binds to mucin-16 (MUC16) to facilitate the migration and metastatic dissemination of PDAC cells (46, 47).

Recent transcriptomic studies found MSLN was associated with anti-tumor immunity. Studies in ovarian cancer and colorectal cancer demonstrated an association between high *MSLN* expression and an immunosuppressive tumor microenvironment (TME) (48, 49). In PDAC, high *MSLN* expression was associated with an increased stromal *CD274* (PD-L1) expression in classical B and basal-like subtypes, which could play a role in immune evasion (50, 51). Another study found that PDAC tumors with high *MSLN* expression had decreased infiltration scores of immune cell subsets (CD4 T cells, CD8 T cells, B cells, and dendritic cells) (36). These findings warrant further characterization of the PDAC tumor landscape to understand the role that MSLN plays in immune regulation.

In this study, we evaluated novel associations between MSLN expression patterns, at both transcript and protein levels, with clinical outcomes and the composition of the immune microenvironment in PDAC patients.

## 2 Materials and methods

### 2.1 Immunohistochemical staining and scoring

Human PDAC tissue microarrays (TMAs), comprising 74 PDAC patients and 14 patients with precursor lesion (13 PanIN and 1 IPMN), were obtained through the Australian Pancreatic Cancer Genome Initiative (APGI) Bioresource (University of Sydney Human Research Ethics Committee: 2018/730). Serial human PDAC formalin-fixed paraffin-embedded (FFPE) sections were also obtained from 10 patients from the Royal Prince Alfred Hospital (RPA) (Sydney Local Health District Human Ethics Committee: 2020/ETH02321). MSLN IHC staining (clone MN-1, Rockland Immunochemicals, Pottstown, PA, USA) and analysis by H-score were undertaken, as previously described (34). Additionally on the serial FFPE sections, CD3 (clone LN10, Novocastra, Leica Microsystems, Deer Park, IL, USA), CD8 (clone C8144B, Dako, Santa Clara, CA, USA), and CD68 (clone KP1, Dako, Santa Clara, CA, USA) IHC staining were undertaken along with routine haematoxylin and eosin (H&E) staining. CD3, CD8, and CD68 scores were evaluated as percentage of stained cells within the tumor stromal area, as previously described (52). Two pathologists independently evaluated the staining, with final scores calculated as the average of their individual assessments.

### 2.2 Transcriptomic data preprocessing

Human PDAC bulk RNA-seq data was obtained from the European Genome-phenome Archive (EGA) database (International Cancer Genome Consortium (ICGC): DACO-7197). Two datasets containing 97 samples (ICGC PACA-AU; EGAD00001003298) and 219 samples (ICGC PACA-CA; EGAD00001003945) were included for analysis. To standardize read alignments across datasets, BAM files were converted to FASTQ using bedtools (ver.2.30.0) (53), and then realigned to the human genome assembly (GENCODE, release 35, GRCh38.p13) using STAR aligner (ver. 2.7.1a) (54). Raw gene counts were enumerated via featureCounts (ver.2.4.2) (55). Batch effects were corrected using the Combat_seq function from sva (ver. 3.50.0) and only counts from protein-coding genes defined by the Human Genome Organisation Gene Nomenclature Committee (HGNC) were retained for analysis (56). Patients with missing clinical information were excluded as well as those with a diagnosis not classified as PDAC.

Mouse PDAC bulk RNA-seq data (n = 37 samples), from a published study (57), were obtained from the Gene Expression Omnibus (GEO) database (GSE109933). Raw read count data was filtered to remove non-protein-coding genes. Seven samples with unknown T cell infiltration status were excluded from the analysis.

Human PDAC single-cell RNA-seq (scRNA-seq) data were sourced from a published study (58). Data from 24 samples were collected as normalized gene expression matrices (Cancer Single-Cell Expression Map (CancerSCEM): https://ngdc.cncb.ac.cn/cancerscem/downloads), on the Genome Sequence Archive (CNCB-NGDC; PRJCA001063). Filtering was performed to retain only high-quality cells, as defined by cells with ≥ 500 detectable genes, ≥ 1500 unique molecular identifiers (UMI), > 0.8 cell complexity (log10 genes per UMI), and <10% of transcripts from mitochondrial genes.

### 2.3 Bulk RNA-seq data analysis

Normalization of raw gene counts and differential expression analysis were conducted via DESeq2 (ver. 1.38.2) (59). For the human dataset, samples in the top and bottom tertiles of *MSLN* expression were compared. Due to smaller sample size (n = 30), the mouse dataset was split based on median *Msln* expression and compared. Upregulated and downregulated genes were identified based on significance (adjusted P-value < 0.05) and expression changes (absolute log2 FC > 0.58). Over-representation analysis of upregulated and downregulated genes was conducted separately via Gene Ontology (GO) enrichment analysis in clusterProfiler (ver 4.7.1.003) (60). Results were visualized using the treeplot function via enrichplot (ver 1. 18.4).

Tumor reactivity was evaluated for human and mouse datasets using the tumor reactive gene signatures (TRS) derived from a previous study, which has been validated in melanoma and several other solid tumor datasets (61). For the mouse dataset, TRS genes were converted to mouse Ensembl IDs. TRS scores were calculated using GSVA (ver 1.46.0) with default parameters as previously described (62).

For estimates of cell type proportions, gene expressions from the human dataset were converted into Transcripts Per Million (TPM) values and analyzed using the “Immune Estimation” algorithm from TIMER2.0 (63).

### 2.4 scRNA-seq analysis

Integration, clustering, and dimensionality reduction of scRNA-seq samples were performed via Seurat (ver 4.3.0) (64). Elbow plots were used to determine the optimal number of principal components (PCs), and PCs 1 to 30 were used for clustering at resolution = 0.5. Annotation was performed at single cell level via SCINA (ver 1.2.0) (65), using cell type identification markers in the original study from which the data was derived (58). Marker expression in each cell type was verified after cell annotation. Samples were assigned to high and low MSLN expression groups based on median cutoff of *MSLN* normalized counts per cell. For analysis of specific subtypes within annotated cell types, cell populations were isolated from the integrated dataset and re-clustered at optimal resolution determined from a range of 0.5, 0.1, and 0.05. Manual annotation was performed for each cluster based on the expression of representative markers, which were identified using the FindAllMarkers function from Seurat (ver 4.3.0). UMAP (Uniform Manifold Approximation and Projection) plots were generated to illustrate cell clusters and specific marker expression across clusters using the DimPlot and FeaturePlot functions from Seurat (ver 4.3.0), respectively. A balloon plot of *MSLN* expression in annotated cell types across samples was generated using the ggballoonplot function in ggpubr (ver 0.6.0). For the macrophage population, M1 and M2 polarization scores were evaluated for each sample via UCell (ver 2.10.1), based on previously established M1 and M2 gene signatures (66, 67).

Differential gene expressions of CD8 T cell clusters from the MSLN-high and MSLN-low groups were assessed using the FindMarkers function based on default thresholds (adjusted P-value < 0.05 and absolute log2 FC > 0.25). Upregulated and downregulated genes were used in downstream GO enrichment analysis and visualized. Phenotypic profiling was performed using ProjecTILs (ver 3.5.1), with phenotypes inferred by projecting CD8 T cells onto the reference atlas of tumor-infiltrating CD8 T cells provided within the package (68). Cytokine signaling activities in CD8 T cells from each sample were evaluated using the CytoSig database via scaper (ver 0.2.0) (69). Expression levels of memory and exhaustion markers, as well as all chemokine and chemokine receptors, were averaged for CD8 T cells from each sample and compared between the MSLN-high and MSLN-low groups.

### 2.5 Statistical analysis

Statistical analysis was performed in GraphPad Prism (ver 10.4.1, San Diego, California, USA) and R Statistical Software (ver 4.4.2, Vienna, Austria). Clinicopathological characteristics associations with MSLN expression from TMA data and RNA-seq data were evaluated using the Mann-Whitney U test for continuous variables, and the chi-squared test for categorical variables. Survival data was analyzed using Kaplan-Meier curves with the log-rank test. Optimal H-score cutoff for survival using the exact distribution of maximally selected rank statistic was used, as previously described (70), using the surv_cutpoint function from survminer (ver 0.5.0). Univariate and multivariate analyses were performed using Cox proportional hazards regression models for estimating hazard ratios (HR) with 95% confidence intervals (CIs). For multivariate analysis, effects of covariates (age, sex, and tumor stage) were accounted for when evaluating survival differences. Unpaired student’s t-test and Pearson correlation analysis were used in other comparisons between two groups of continuous variables. In all cases, two-tailed tests were used, and statistical significance was set at p < 0.05.

## 3 Results

### 3.1 High MSLN is associated with increased relapse-free survival

**Figure 1:**
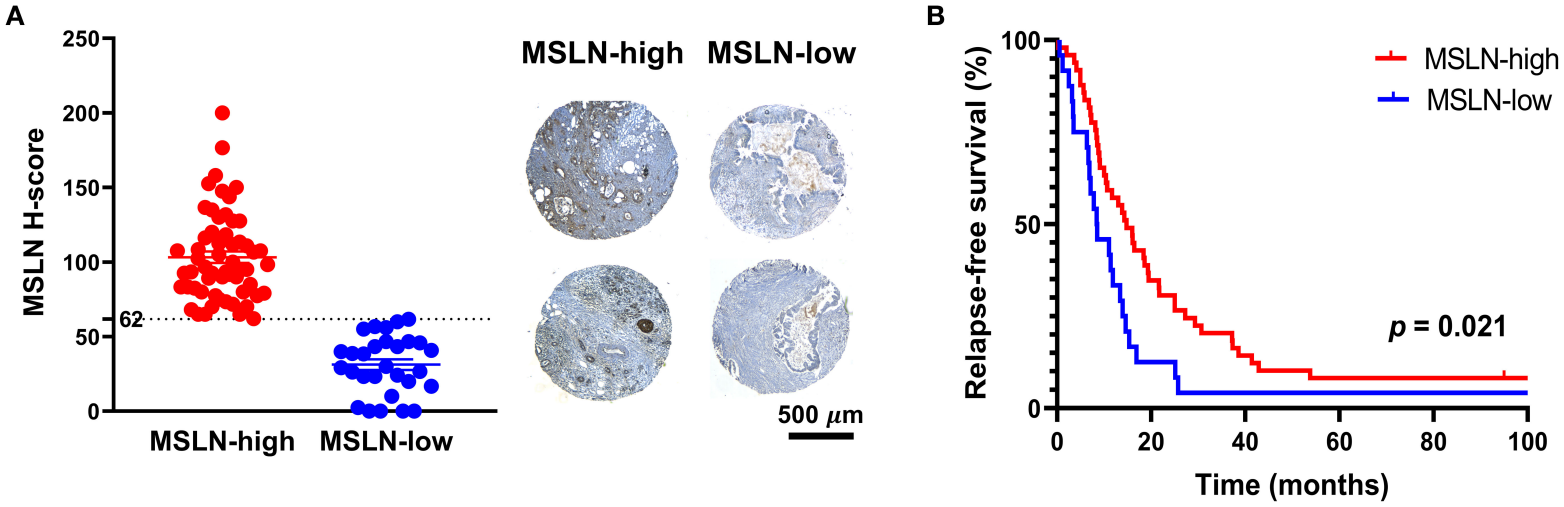
Expression and prognostic value of mesothelin (MSLN). **(A)** Distribution of MSLN expression across the cohort based on H-score cutoff of 62 (dotted line) (left). Mean ± SEM. Representative images of tissue microarray samples from the MSLN-high and MSLN-low groups (right). Scale bar = 500 µm. **(B)** Kaplan Meier curves of relapse-free survival of MSLN-high and MSLN-low groups. P-value was derived from log-rank test.

The clinical characteristics of the 74 PDAC TMA patients are summarized in **Table 1**. Twelve of the patients (16.2%) received chemotherapy, as adjuvant (n = 7), neoadjuvant (n = 2), and/or palliative (n = 3) treatment. No difference in MSLN H-score was observed between the PDAC and the precursor lesion cohorts (14 patients consisting of PanINs and IPMNs) (**Supplementary Figure S1A**). Using an H-score cutoff of 62 (**Supplementary Figure S1B**), 32% (n = 24) were classified as MSLN-low and 68% (n = 50) were classified as MSLN-high (**Figure 1A**). The MSLN-high group had significantly higher relapse-free survival (RFS) with a median of 14.5 months (95% CI = 10.0 – 21.6 months), compared to a median RFS survival of 8.5 months (95% CI = 6.9 – 13.9 months) in the MSLN-low group (p = 0.021) (**Figure 1B**). The MSLN-high group had significantly reduced univariate HR (0.571, 95% CI = 0.343 – 0.951, p = 0.031) and reduced, albeit not significant, HR by multivariate analysis, adjusted for age, sex, and tumor stage (0.618, 95% CI = 0.332 – 1.147, p = 0.127). Clinicopathological associations with MSLN expression found that the MSLN-high group exhibited a positive association with increased age (p = 0.036) (**Table 2**). There was no difference between MSLN expression with respect to all other parameters examined including sex, tumor characteristics (stage, size, location, differentiation, and residual tumor), invasion (in peritoneum, and vasculature) and lymph node involvements.

**Table 1:** Demographic and clinicopathological summary of Australian PDAC patients in the tissue microarray cohort.

Parameter	Patient *n* (%)
Total	74 (100.0)
Age (years)	74 (100.0)
< 65	36 (48.6)
≥ 65	38 (51.4)
Sex	74 (100.0)
Male	39 (52.7)
Female	35 (47.3)
Tumor stage	72 (97.3)
IA	3 (4.2)
IB	8 (11.1)
IIA	20 (27.8)
IIB	38 (52.8)
IV	3 (4.2)

**Table 2:** Associations between mesothelin expression levels and clinicopathological characteristics of Australian PDAC patients in the tissue microarray cohort.

Parameter	Category	Total (*n* = 74)	MSLN-high (*n* = 50)	MSLN-low (*n* = 24)	P-value
Age (years), median (range)		65 (40-83)	66.5 (44-79)	60.5 (40-83)	0.036
Sex, *n* (%) 74 (100.0)	Male	39 (52.7)	29 (58.0)	10 (41.7)	ns
	Female	35 (47.3)	21 (42.0)	14 (58.3)	
Tumor stage, *n* (%) 72 (97.3)	IA-IB	11 (15.3)	9 (18.4)	2 (8.7)	ns
	IIA	20 (27.8)	13 (26.5)	7 (30.4)	
	IIB - IV	41 (56.9)	27 (55.1)	14 (60.9)	
Tumor size, *n* (%) 64 (86.5)	≤ 3.5 cm	42 (65.6)	31 (68.9)	11 (57.9)	ns
	> 3.5 cm	22 (34.4)	14 (31.1)	8 (42.1)	
Tumor location, *n* (%) 55 (74.3)	Head	42 (76.4)	28 (73.7)	14 (82.4)	ns
	Body	4 (7.3)	3 (7.9)	1 (5.9)	
	Tail	9 (16.4)	7 (18.4)	2 (11.8)	
Tumor differentiation, *n* (%) 72 (97.3)	Well differentiated	8 (11.1)	6 (12.5)	2 (8.3)	ns
	Moderately differentiated	41 (56.9)	27 (56.3)	14 (58.3)	
	Poorly differentiated	23 (31.9)	15 (31.3)	8 (33.3)	
Residual tumor, *n* (%) 57 (77.0)	No residual tumor	32 (56.1)	18 (52.9)	14 (60.9)	ns
	Residual microscopic tumor	25 (43.9)	16 (47.1)	9 (39.1)	
Peritoneal invasion, *n* (%) 54 (73.0)	Absent	9 (16.7)	8 (20.0)	1 (7.1)	ns
	Present	45 (83.3)	32 (80.0)	13 (92.9)	
Vascular invasion, *n* (%) 35 (47.3)	Absent	14 (40.0)	11 (42.3)	3 (33.3)	ns
	Present	21 (60.0)	15 (57.7)	6 (66.7)	
Lymph nodes involved, *n* (%) 59 (79.7)	0	26 (44.1)	19 (47.5)	7 (36.8)	ns
	1-3	24 (40.7)	16 (40.0)	8 (42.1)	
	4-7	9 (15.3)	5 (12.5)	4 (21.1)	

MSLN, mesothelin; ns, not significant.

Interestingly, no significant difference in patient outcomes (overall and relapse-free survival) was observed in relation to MSLN expression levels in the RNA-seq datasets (**Supplementary Figure S2**). The MSLN-high group did not correlate with any of the clinicopathological parameters examined, including age, sex, tumor characteristics (stage, location, and differentiation), treatment type, response, and relapse status (**Supplementary Table S1**). This discrepancy could be due to differences in MSLN expressions at transcript versus protein levels.

### 3.2 High MSLN is associated with reduced immune activity in human and mouse PDAC tumors

**Figure 2:**
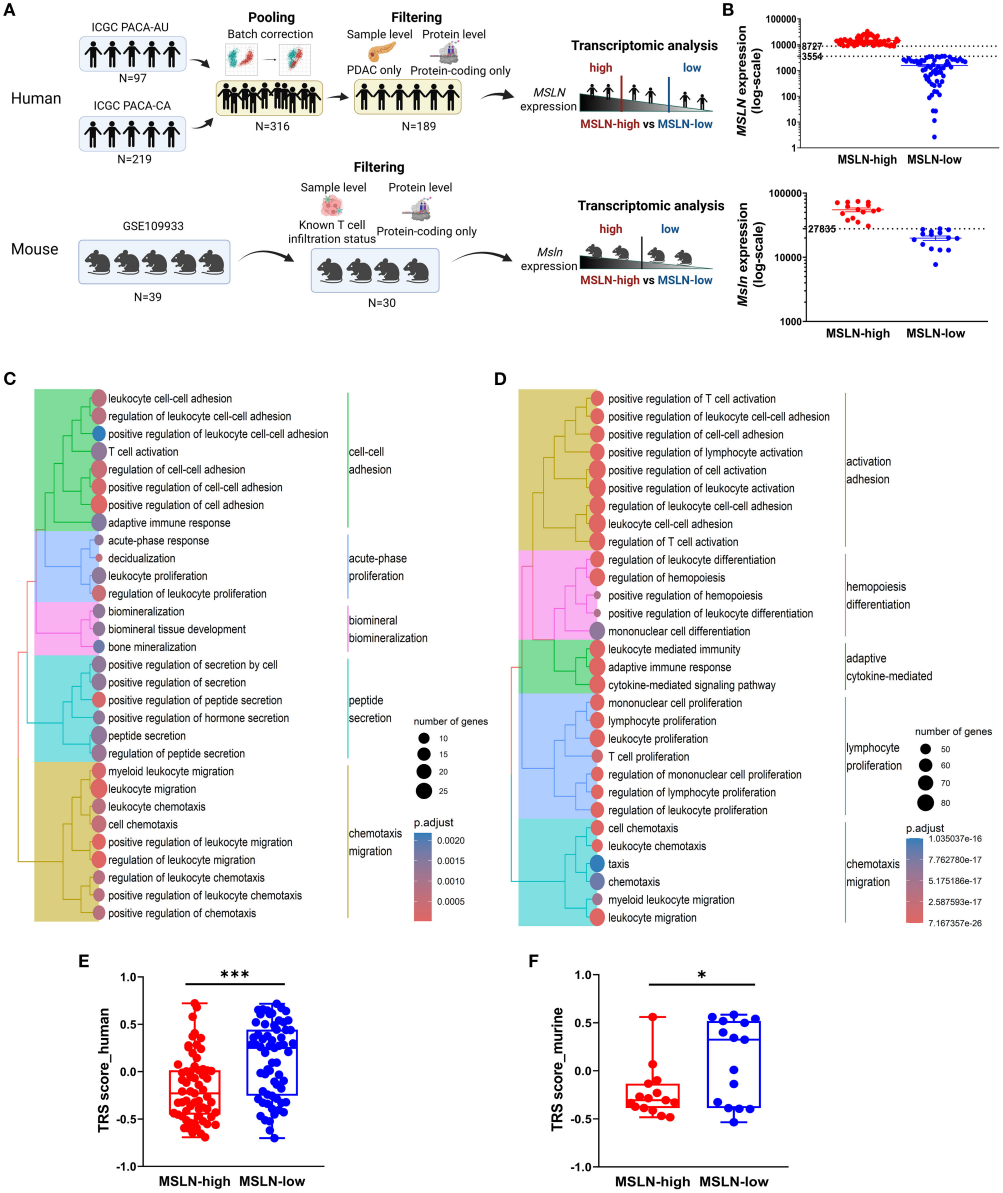
High mesothelin (human: *MSLN*, mouse: *Msln*) transcript is associated with decreased immune functions and tumor reactivity. **(A)** Workflow for transcriptomic analysis of human (top) and mouse (bottom) bulk RNA-sequencing datasets. **(B)** Human (top) and mouse (bottom) datasets were separated into MSLN-high and MSLN-low groups based on transcript expression of MSLN. Expression was quantified as normalized read counts (DESeq2). Expression thresholds for stratification are indicated (dashed lines). Top 30 biological processes from gene ontology enrichment analysis of downregulated genes in MSLN-high vs MSLN-low groups from the human **(C)** and mouse **(D)** datasets. Predicted tumor-reactive T cell signatures (TRS) scores for MSLN-high and MSLN*-*low groups from the human **(E)** and mouse **(F)** datasets. Statistical testing by student’s t-tests (*p < 0.05; ***p < 0.001; ns, not significant).

To investigate the biological significance of MSLN, transcriptomic analysis was conducted on human and mouse RNA-seq datasets to compare samples with high and low MSLN expressions (**Figures 2A, B**). In both datasets, the MSLN-high group exhibited downregulation of genes involved in immune-associated pathways, including the regulation of leukocyte adhesion, proliferation, and migration/chemotaxis (**Figures 2C, D**). In addition, T cell activation and more broadly adaptive immune response pathways were downregulated. Within the top 30 downregulated pathways examined, the human RNA-seq dataset also included two clusters of pathways participating in bone development and peptide secretions, although these were not observed in the mouse RNA-seq dataset, which was comprised only of immune-associated clusters. To examine anti-tumor responses, tumor reactivity was predicted using tumor reactive CD8 T cell signature (TRS) scores from a previous study (61), which has been validated using hepatocellular carcinoma, non-small-cell lung cancer, melanoma, and colorectal cancer datasets. The MSLN-high group in both human and mouse datasets showed significantly lower TRS scores, indicating high MSLN expression is potentially associated with reduced anti-tumor immune responses (**Figures 2E, F**).

To investigate if immune cell infiltration into tumors also decreased, the relative proportions of key immune cell infiltrates (such as T cells and macrophages) were estimated via cell type prediction algorithms and compared between the MSLN-high and MSLN-low groups in the human RNA-seq dataset. However, strong discordance was observed across the algorithms (**Supplementary Figure S3**). For the mouse RNA-seq dataset, T cell infiltration status of the implanted tumor clones, described in the study from which the mouse data was derived (57), was not associated with *Msln* expression. *Msln* expression did not differ significantly between “T cell high” and “T cell low” clones, nor did tumors in the MSLN-high group have higher proportions of “T cell high” clones (**Supplementary Figure S4**).

### 3.3 Cytotoxic T cells are reduced in PDAC tumors with high *MSLN* expression by single-cell RNA-seq

**Figure 3:**
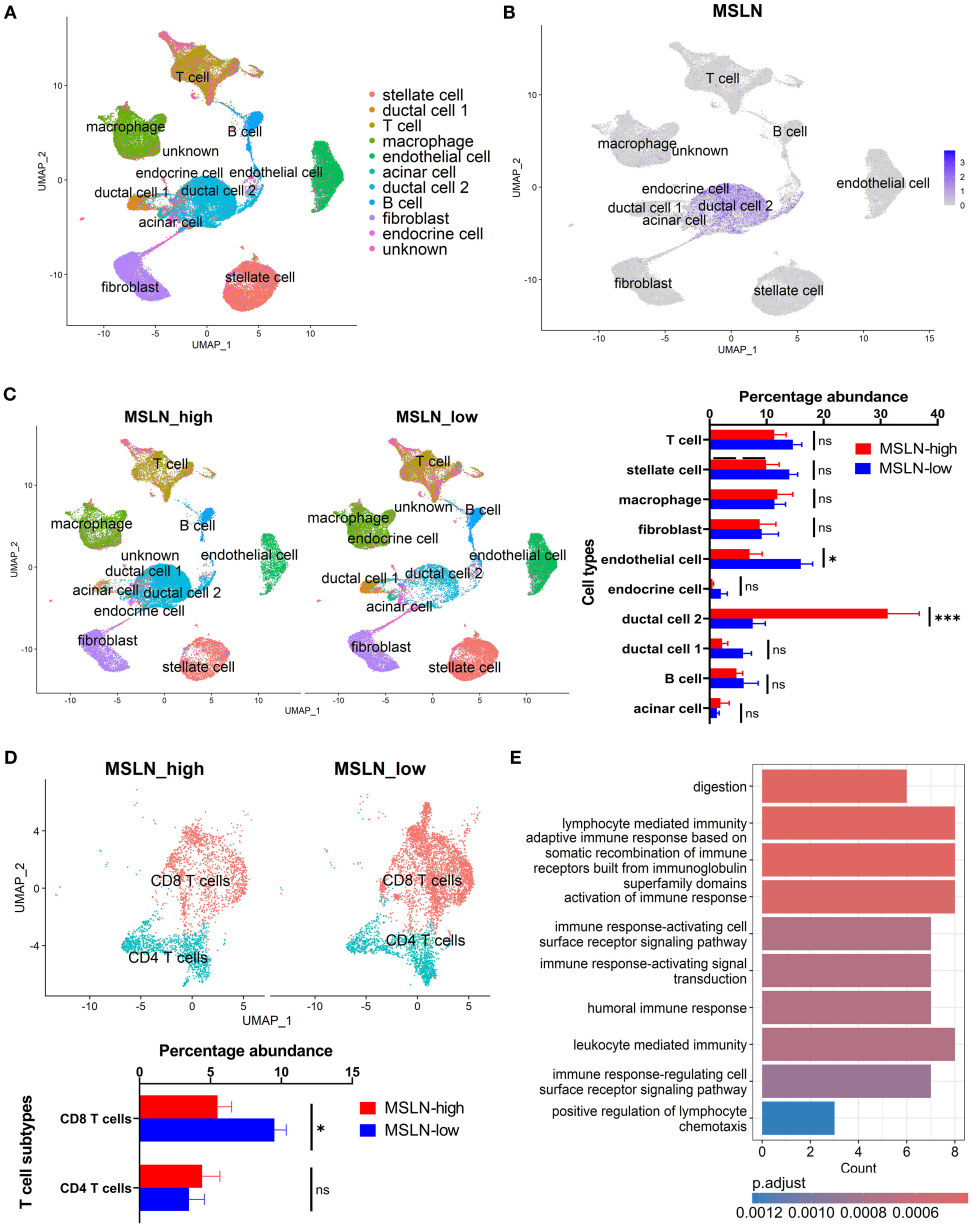
Characterization of human pancreatic cancer from single-cell transcriptomics based on mesothelin (*MSLN*) expression. **(A)** UMAP visualization showing the clustering of cells following integration of all samples. Cell type annotations represented by different colors. **(B)** Feature plot indicating the distribution of *MSLN* expression across the annotated cell clusters. Color scale shows the level of *MSLN* expression, with higher intensity indicating higher expression. **(C)** Comparison of the profiles of annotated cell types in MSLN-high and MSLN-low groups. Samples were stratified based on median *MSLN* normalized counts per cell. Overall landscape based on UMAP visualization (left) and quantified differences in percentage abundance (percentage out of total cells) of cell types (right). **(D)** T cell subtype profiles in the MSLN-high vs MSLN-low groups. **(E)** Bar plot of the top 10 downregulated biological processes from gene ontology analysis of CD8 T cells in MSLN-high vs MSLN-low groups. Mean ± SEM. Statistical testing by student’s t-tests (*p < 0.05; ***p < 0.001; ns, not significant).

In the human scRNA-seq dataset, *MSLN* expression was mainly distributed in a malignant ductal 2 cell population, as demonstrated previously (58) (**Figures 3A, B**). Highest *MSLN* expression was also confirmed in the ductal 2 cells based on the intensity of expression and percentage abundance (percentage out of total cells) in individual samples (**Supplementary Figure S5**). The MSLN-high group exhibited a significantly higher percentage of ductal 2 cells (mean ± SEM: 31.2% ± 5.5% vs 7.6% ± 2.2%) and lower percentage of endothelial cells (mean ± SEM: 7.0% ± 2.2% vs 16.0% ± 2.1%) (**Figure 3C**). Further characterization revealed a *MUC1*-positive cluster to be the predominant subtype of ductal 2 cells, but both the *MUC1*-positive and one of the *MUC1*-negative clusters showed increased percentages abundance in the MSLN-high group (**Supplementary Figure S6A**). The endothelial cells were comprised of three clusters representing an arterial population and two (*PLVAP*+/*POSTN*+) venous populations (**Supplementary Figure S6B**). Reductions in percentage abundance were observed only in the *PLVAP*+ venous subtype, which comprised the majority (~65%) of endothelial cells in MSLN-high vs MSLN-low groups.

Although differences in global abundance of T cell infiltrates were not observed, the CD8 T cell subset showed significantly reduced percentage abundance in the MSLN-high group (mean ± SEM: 5.5% ± 1.0% vs 9.5% ± 0.8%) (**Figure 3D**). This represents a more than 40% reduction in total CD8 T cell populations, when compared to the MSLN-low group. Other immune subsets (CD4 T cells, B cell and macrophage subsets) did not show any significant difference in percentage between MSLN-high and MSLN-low groups (**Supplementary Figures S6C, D**). However, within the macrophage population, the MSLN-high group demonstrated a shift towards an M2-polarized phenotype (**Supplementary Figure S7**).

Transcriptomic profiles of CD8 T cell subset in the MSLN-high group showed genes involved in immune-associated activity pathways to be downregulated compared to the MSLN-low group (**Figure 3E**). These pathways participate in adaptive immune responses, immune activation, and chemotaxis, consistent with the bulk RNA-seq analysis. The memory and exhaustion phenotypes, as well as cytokine and chemokine profiles, of CD8 T cells were further characterized. No significant differences were observed in the memory or exhaustion phenotypes between MSLN-high and MSLN-low groups (**Supplementary Figure S8**). When compared to the MSLN-low group, CD8 T cells from MSLN-high group showed enrichment of GMCSF, HGF, IL-1, IL-2, and TNFSF12 signaling pathways (**Supplementary Figure S9**). These cells also showed downregulated expressions of chemokines *CCL2*, *XCL1*, and *XCL2*, as well as the chemokine receptor *CXCR6* (**Supplementary Figure S10**). However, expression of *CXCL5*, a neutrophil chemoattractant known to impair CD8 T cell-mediated anti-tumor immunity (71), was upregulated. These findings suggest that high *MSLN* expression is associated with reduced abundance and altered transcriptomic activities of CD8 T cell infiltrates in PDAC.

### 3.4 Tumors with high MSLN expression show reduced cytotoxic T cell infiltration

**Figure 4:**
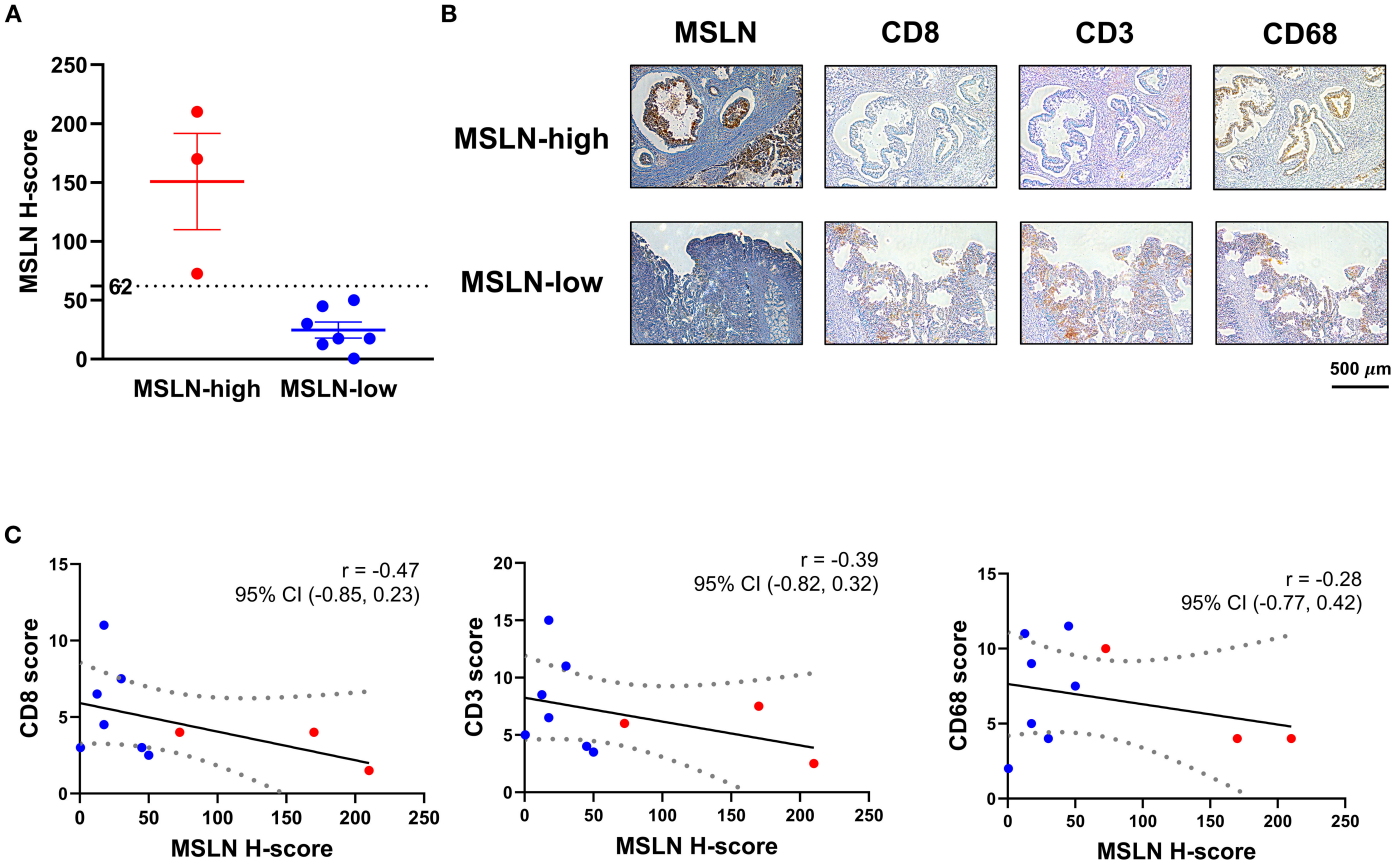
Associations of mesothelin (MSLN) expression with T cell infiltration in tumor stroma. **(A)** H-score distribution of MSLN staining on tissue sections from surgical specimens. Samples were stratified into MSLN-high and MSLN-low groups based on the H-score of 62. **(B)** Immunohistochemical (IHC) staining of representative serial FFPE sections from samples in the MSLN-high and MSLN-low groups. Scale bar = 500 µm. **(C)** Correlational analysis of MSLN H-score with CD8, CD3, and CD68 scores. Linear regression model was fitted (solid line), with dashed boundaries representing the 95% confidence interval (CI). Samples classified as MSLN-high (red) and MSLN-low (blue) groups were highlighted. Correlation coefficient (r) and its 95% CI were indicated for each pair-wise analysis.

To validate the transcriptomic relationship between MSLN expression and T cell infiltration, IHC staining on 10 surgically resected PDAC tumors was undertaken. MSLN expression (evaluated as H-score) showed a range from 0.5 – 210 (**Figure 4A**). Using the H-score cutoff of 62, the MSLN-high group (n=3) exhibited less intense, albeit not significant, staining of CD8 (mean ± SEM: 3.167 ± 0.833 vs 5.429 ± 1.172, p = 0.272) and CD3 (mean ± SEM: 5.333 ± 1.481 vs 7.643 ± 1.580, p = 0.409) in the tumor stroma (**Figure 4B**; **Supplementary Figure S11**). CD68, used as a negative control, showed comparable staining between MSLN-high and MSLN-low groups (mean ± SEM: 6.000 ± 2.000 vs 7.143 ± 1.366, p = 0.656). Across all samples, a decreasing trend was observed in all three (CD8, CD3, and CD68) scores with increasing MSLN H-score, but correlations did not reach significance (CD8: p = 0.174; CD3: p = 0.267; CD68: p = 0.432), likely due to low sample numbers (**Figure 4C**). Overall, a decreased trend in CD8 T cell infiltration was observed in MSLN-high tumors.

## 4 Discussion

This study identified high MSLN expression (H-score ≥ 62 from IHC staining) in PDAC to be associated with improved RFS and age. Transcriptomic analysis found a link between MSLN expression and an immunosuppressive tumor landscape. Specifically, CD8 T cells had reduced immune reactivity and reduced percentage abundance in PDAC tumors with high MSLN expression. In subsequent IHC validation, PDAC tumors with high MSLN expression demonstrated reduced infiltration of CD8 T cells in the stroma, although significance is not reached and confirmations in larger independent cohorts remain necessary.

The study identified MSLN as a biomarker for improved prognosis, which contrasts previous studies that found high MSLN expression to be correlated with worse survival outcome in PDAC (37, 38, 46). This discrepancy could be due to the different methodological classification and scoring used. Only one other study in PDAC used the H-score system for stratification. Using a median MSLN H-score cutoff of 180, they found poor survival in patients with high co-expression of MSLN and MUC16 (46). Other studies established cutoffs either based on the percentage of MSLN-positive cells alone (38) or the percentage of positive cells with the staining intensity analyzed separately (37). Antibody clones for MSLN staining also varied in studies. Two studies used anti-MSLN antibody clone 5B2 (37, 46), in contrast to the MN-1 clone used in the current study. The 5B2 clone has been found to have lower affinity and staining positivity in PDAC compared to the MN-1 clone (72). Staining patterns also differ between MN-1 and 5B2 clones, likely due to differential expression of epitopes for MSLN recognition, where the exact binding site for 5B2 has not been characterized (34).

Underlying cohort-specific factors can potentially contribute to the observed findings as well. Our TMA cohort is relatively small (n = 74), with only a limited number of individuals receiving adjuvant chemotherapy (n = 7) and having available resection margin data (n = 56). Consequently, the effects of surgical resection and adjuvant chemotherapy on RFS could not be comprehensively examined in this cohort and were therefore excluded from the multivariate analysis, although they may represent potential confounders. Further investigation in a larger cohort and with biopsy samples are warranted.

Our study is the first to examine MSLN expression in an Australian PDAC population via IHC. Interestingly, high MSLN expression, in tumors of an Australian mesothelioma patient cohort, was also associated with improved patient outcomes (34). In mesothelioma, the epithelioid subtype shows higher MSLN expression and a more favorable prognosis than the less differentiated sarcomatoid and biphasic subtypes (73). Although MSLN was not associated with the histological grade (**Table 2**), the relationship of MSLN expression with molecular subtypes of PDAC have not been examined in this Australian cohort, due to the lack of patient-matched transcriptomic data, and requires further investigation. Additionally, multiple proteases in the ADAM, MMP, and BACE families have been known to shed MSLN from cancer cells (74). Tumors with high MSLN expression could potentially be more resistant to antigen shedding, thus enabling greater surface antigen availability for immune surveillance, as MSLN-specific CD4 and CD8 T cells have been detected in the peripheral circulation of PDAC patients (75). Conversely, tumors with low cell-surface MSLN expression and high shedding activity may release elevated levels of soluble MSLN into the circulation, where sustained exposure could contribute to T cell anergy over time (76), potentially leading to poorer prognosis. Notably, MSLN shedding and other post-translational processing such as antigen maturation may result in discrepancies of MSLN expression at the RNA and protein levels, hence possibly explaining the different prognostic outcomes from the IHC and bulk RNA-seq data. Further validation using an independent Australian cohort is needed to determine whether the positive prognostic value of MSLN is reproducible and reflects a generalizable biological phenomenon or is influenced by population-specific genetic and/or environmental factors. The Australian population is racially and ethnically diverse and a comparison with other populations could be of interest.

Our finding that MSLN expression is associated with an immunosuppressive microenvironment is consistent with previous RNA-seq analyses (36, 50). In one study, a positive correlation between tumor *MSLN* expression and stromal *CD274* (PD-L1) expression was found using the deconvoluted ICGC RNA-seq data and validated *in vitro* (50). PD-L1, upon binding to the PD-1 receptor, is known to suppress T cell activating signals and inhibit anti-tumor responses (77). Although our study did not directly examine PD-1/PD-L1 signaling pathways, transcriptomic analyses of both mouse and human RNA-seq datasets revealed that MSLN-high tumors exhibited decreased T cell activation signatures and suppressed tumor reactivity scores. However, in scRNA-seq, exhaustion phenotypes of CD8 T cells did not show significant differences between MSLN-high and MSLN-low groups. Downregulation of other immune-related pathways (such as leukocyte adhesion, proliferation and chemotaxis) was also observed in this study and suggests that additional immunosuppressive mechanisms could exist in MSLN-high tumors. In particular, we confirmed that expressions of chemokines and chemokine receptors that promote T cell migration and anti-tumor activities were suppressed in CD8 T cells from MSLN-high tumors, whereas expression of the immunosuppressive cytokine, CXCL5, was elevated. Furthermore, a reduced proportion of endothelial cells in the *PLVAP+* venous subtype was observed in the scRNA-seq dataset. PLVAP is known to regulate vascular permeability and facilitates leukocyte trafficking (78–80). Thus, decreased abundance of *PLVAP*+ endothelial cells could be linked to reductions in CD8 T cell infiltration as well.

In ovarian cancer, MSLN activates Wnt/β-catenin signaling to induce protumorigenic macrophage polarization via CD24 upregulation (81). While CD24 upregulation was not observed in our study from both the bulk RNA-seq and scRNA-seq analyses, we did find macrophages in MSLN-high tumors to exhibit increased polarization towards the tumorigenic M2 phenotype. MSLN overexpression has been shown to promote autocrine IL-6 signaling in PDAC cells (44); however, its association with cytokine signaling in T cells has not been specifically investigated. In our scRNA-seq analysis, we observed increased activity of pro-inflammatory cytokine signaling in CD8 T cells from MSLN-high tumors. Notably, this association was not identified in our bulk RNA-seq data, where such upregulated cytokine signaling activity may potentially be obscured by reduced infiltration of CD8 T cells. These suggest that high MSLN expression may be linked to broader immunomodulation within the PDAC TME, while the exact biological pathways underlying the observed functional changes in these immune infiltrates remain to be fully characterized.

High *MSLN* expression has been associated with reduced CD8 T cell infiltration in PDAC tumors in two independent human RNA-seq cohorts (TCGA and GSE62452) (36). Cell type compositions and immune activities were inferred based on the xCell algorithm (82). Although cohort-specific variations in multiple immune cell types, such as dendritic cells, were also observed, only CD8 T cells showed a consistent decrease in both RNA-seq cohorts. Suppressed immune responses (in lymphocyte infiltration, T-cell receptor richness, and cytolytic activity scores) were also associated with high *MSLN* expression. Nevertheless, cell type estimates and immune response predictions remain limited from bulk RNA-seq, as *bona fide* immune cell populations cannot be isolated for independent characterization. In the current study, estimates of cell type compositions from human RNA-seq samples demonstrated large discrepancies across the prediction tools used. Consequently, we confirmed CD8 T cell infiltration by scRNA-seq analysis as well as by IHC staining. Convincingly, as determined by scRNA-seq, CD8 T cells were the only immune subset that exhibited a significant reduction in abundance (~4% of total cells per sample, or 42% of total CD8 population) when comparing MSLN-high to MSLN-low tumors. The IHC validation also found a trend towards reduced CD8 T cell stromal infiltration but did not reach significance, likely due to the small sample size of this exploratory cohort (n = 10). Similarly, assessment of CD8 T cells using the MSLN cutoff defined in Section 3.1 (H-score = 62) showed an overall reduction in the MSLN-high group, but did not reach significance, likely due to the very limited number of cases remaining in this group after stratification (n = 3). The consistent inverse relationship between MSLN expression and CD8 T cell infiltration observed across multiple datasets warrants histopathological validation in larger independent cohorts in future studies.

It remains to be addressed whether there is a causative effect between MSLN expression and immunosuppression in PDAC. Our analysis on mouse RNA-seq data suggested that there was a lack of association between T cell infiltration status of the implanted tumor clones and tumor *Msln* levels. This suggests that immunosuppressive tumors did not cause upregulations of *Msln* expression. These findings, and whether high MSLN expression induces immunosuppression, remain to be tested in human-based experimental models. MSLN expression and CD8 T cell infiltration may also be specific to PDAC. Analyses in other MSLN-expressing tumors, such as mesothelioma, have interestingly indicated an opposite relationship where high MSLN expression was associated with high CD8 T cell density in TMAs (83). Transcriptomic analysis in ovarian and colorectal cancer also found higher CD8 T cell infiltration and higher T cell inflamed score, respectively, despite an overall positive association with an immunosuppressive tumor landscape (48, 49). Further studies to elucidate the mechanisms for MSLN and immuno-modulation are required, and to confirm whether this is a direct causative effect.

In summary, this study investigated the clinicopathological and prognostic significance of MSLN expression in an Australian PDAC cohort. A significant association between high MSLN expression and an immunosuppressive tumor microenvironment was also identified in PDAC, characterized specifically by reduced CD8 T cell infiltration. These findings have important clinical implications for treatment selection. Patients with low MSLN expression may derive greater benefit from immune checkpoint inhibitors (anti-PD-1 and anti-CTLA-4 antibodies) due to their relatively higher baseline CD8 T cell infiltration levels. Conversely, patients with high MSLN expression might be better candidates for MSLN-targeted therapies, such as the SS1P immunotoxin, given their increased target antigen expression. By elucidating the relationship between MSLN expression and immune contexture in PDAC, our work provides a foundation for developing more personalized treatment strategies that may improve patient outcomes.

## Data Availability

The original contributions presented in the study are included in the article/**Supplementary Material**. Further inquiries can be directed to the corresponding author.
