# Supplementary material for: High mesothelin expression is associated with low cytotoxic T cell infiltration in pancreatic cancer
Source: Front Immunol. 2025 Oct 8;16:1651687. doi: 10.3389/fimmu.2025.1651687 (PMC12540403; doi:10.3389/fimmu.2025.1651687)
Supplement: Supplementary file 1 [file DataSheet1.pdf]

Supplementary Material

1 Supplementary Data

Supplementary Data (Supplemental Data 1_Clinicopathological information of the Australian TMA cohort.xlsx and Supplementary Data 2_IHC scores of surgical PDAC FFPE sections.xlsx) have been uploaded separately.

2 Supplementary Figures and Tables

2.1 Supplementary Figures

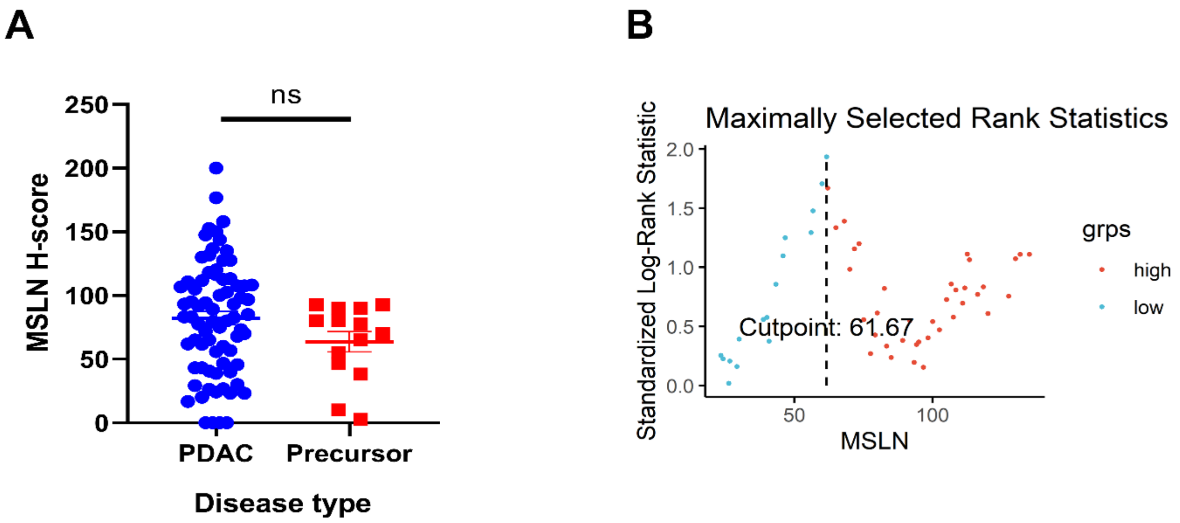

Supplementary Figure 1. Mesothelin (MSLN) expression distribution and determination of optimal H-score cutoff from the tissue microarray cohort. (**A**) MSLN expression, evaluated as H-scores, across pancreatic cancer subtypes – pancreatic ductal adenocarcinoma (PDAC) and pancreatic precursors. Precursor group includes pancreatic intraepithelial neoplasms (PanINs) and intraductal papillary mucinous neoplasms (IPMNs). Statistical testing by student's t-test (ns, not significant). Mean ± SEM. (**B**) In the PDAC group, the optimal H-score cutoff providing the highest standardized log-rank statistics was evaluated based on maximally selected rank statistics and indicated. grps, shorthand for groups.

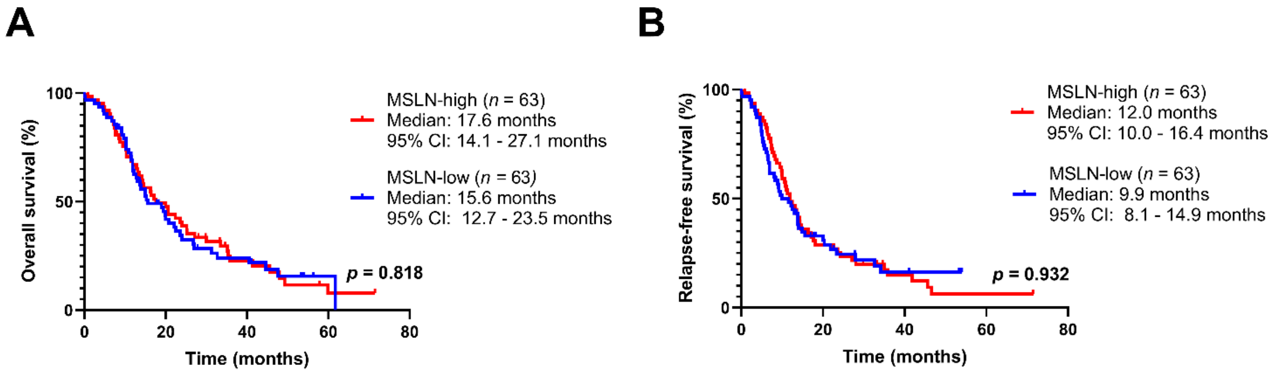

Supplementary Figure 2. Survival differences in the human PDAC RNA-sequencing (RNA-seq) dataset. Top and bottom tertiles of MSLN transcript (*MSLN*) expression were used to establish the MSLN-high and MSLN-low groups. Kaplan-Meier curves of relapse-free survival (RFS) (**A**) and overall survival (OS) (**B**) were shown. Censored events were indicated, along with median survival and 90% confidence interval (CI). P-values were reported from log-rank tests (ns, not significant). PDAC, pancreatic ductal adenocarcinoma.

A

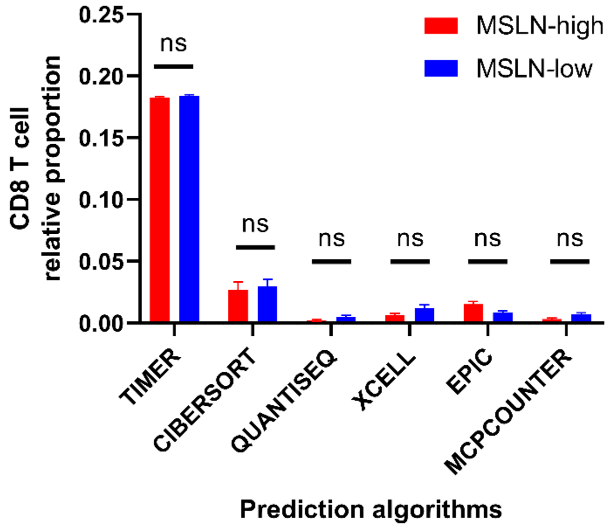

B

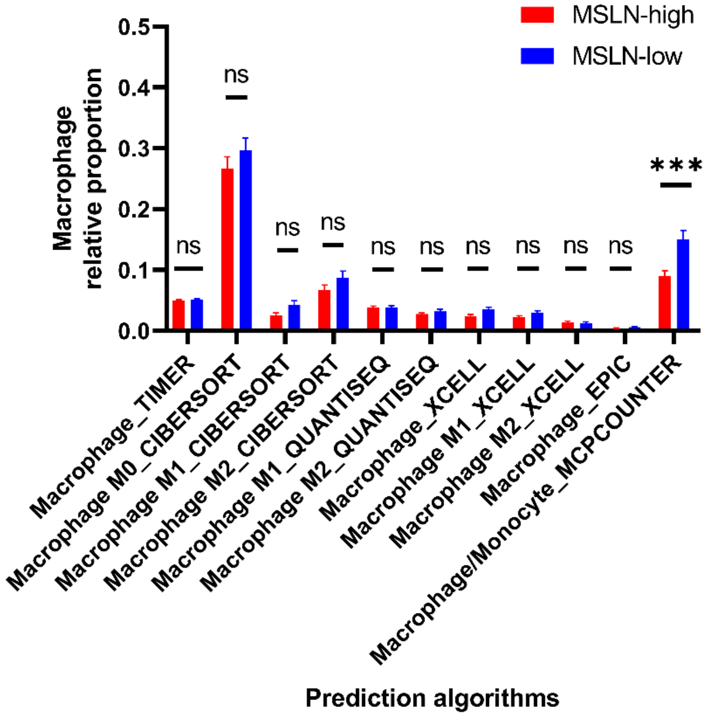

Supplementary Figure 3. Representative examples of cell type proportions estimated using multiple cell type prediction algorithms. Estimated relative proportions of CD8 T cells (**A**) and macrophage populations (**B**) in the MSLN-high and MSLN-low groups of the human RNA-sequencing (RNA-seq) dataset were shown. Mean ± SEM. Statistical testing by student's t-tests (***$p < 0.001$; ns, not significant).

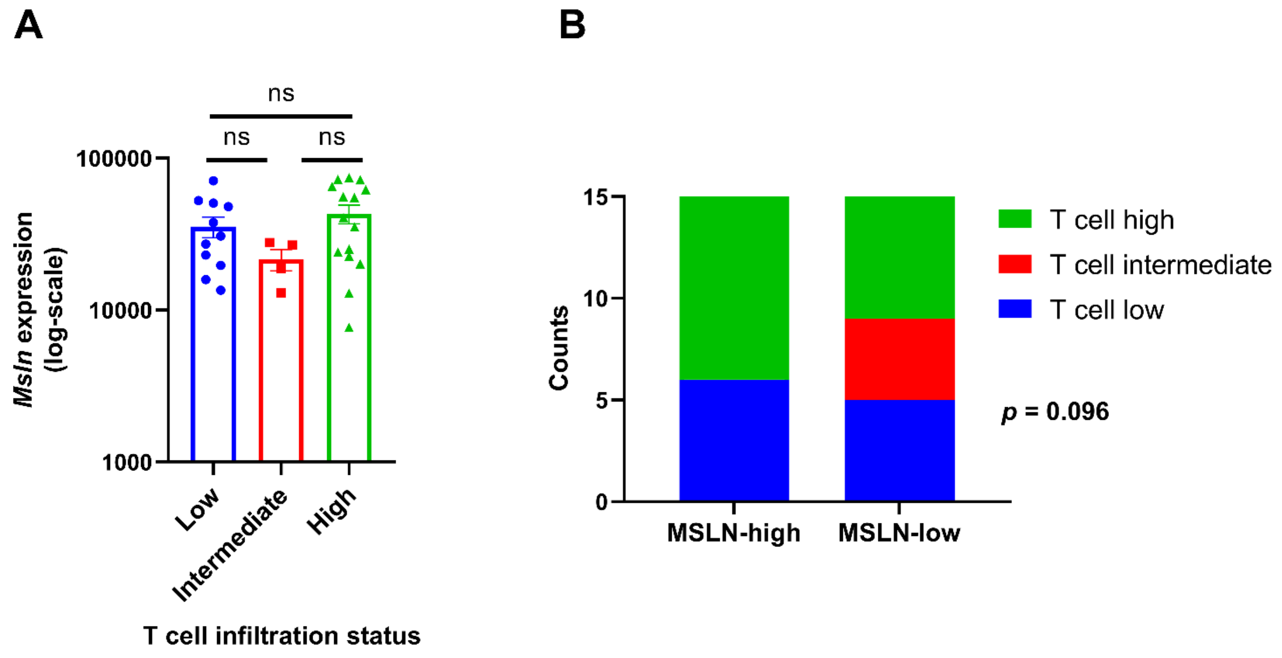

Supplementary Figure 4. Relationships between T cell infiltration status and transcript expression of mouse mesothelin (*Msln*) in the mouse pancreatic cancer RNA-sequencing dataset. (**A**) T cell infiltration status of implanted tumor clones and *Msln* expression in harvested tumor samples. *Msln* expression was quantified as normalized DESeq2 counts and represented on log10 scale. Mean ± SEM. (**B**) Breakdown of samples with different T cell infiltration status in MSLN-high and MSLN-low groups, stratified based on median *Msln* expression. Statistical testing used in (**A**) was student's t-test (ns, not significant) and in (**B**) was chi-squared test. P-value in (**B**) was indicated.

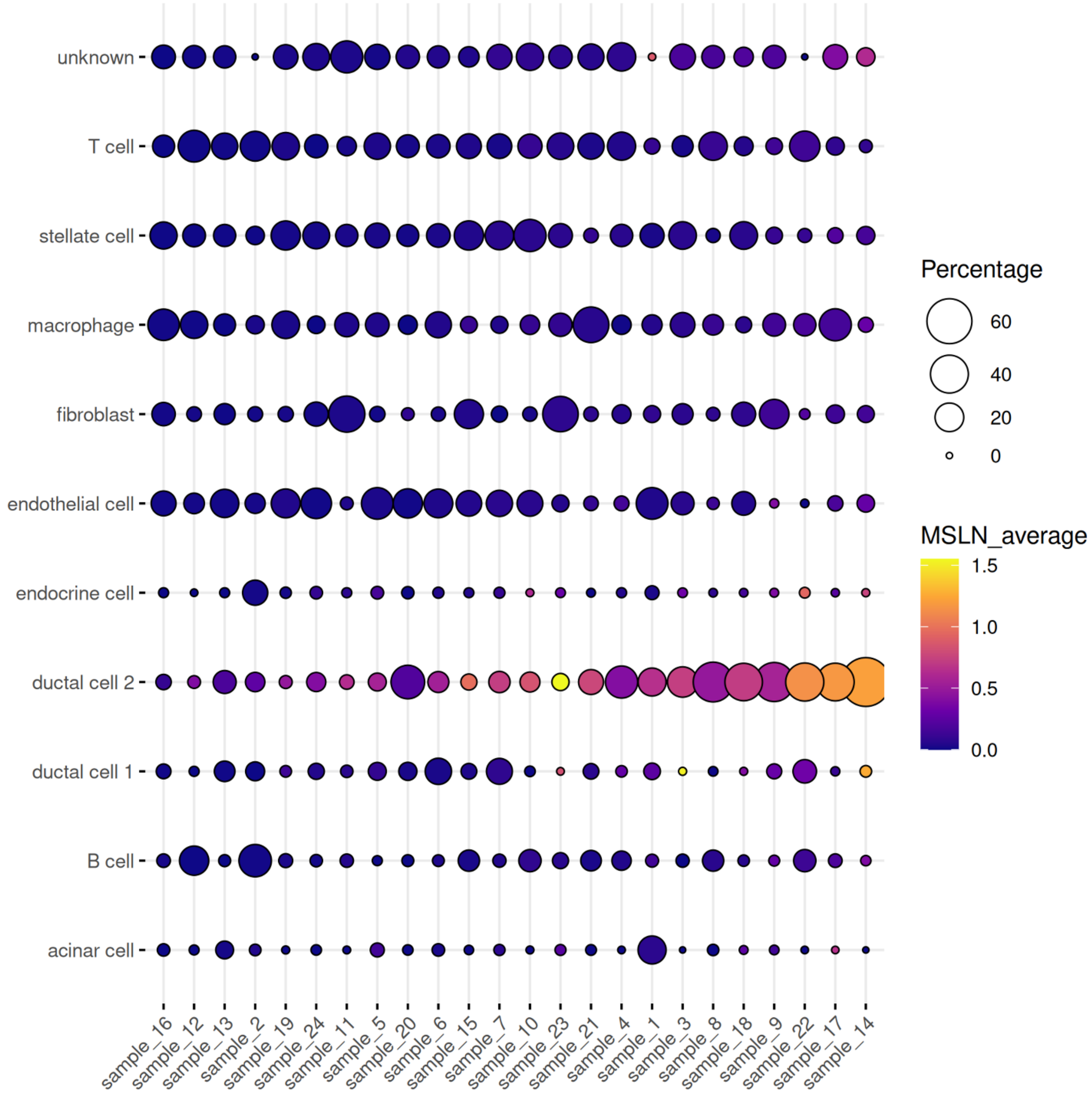

Supplementary Figure 5. Balloon plot showing the average mesothelin transcript (*MSLN*) expression and percentage (%) of the annotated cell types for individual samples in the single cell RNA-sequencing dataset. Samples were ordered based on lowest (leftmost) to highest (rightmost) mean *MSLN* expression across all cell populations.

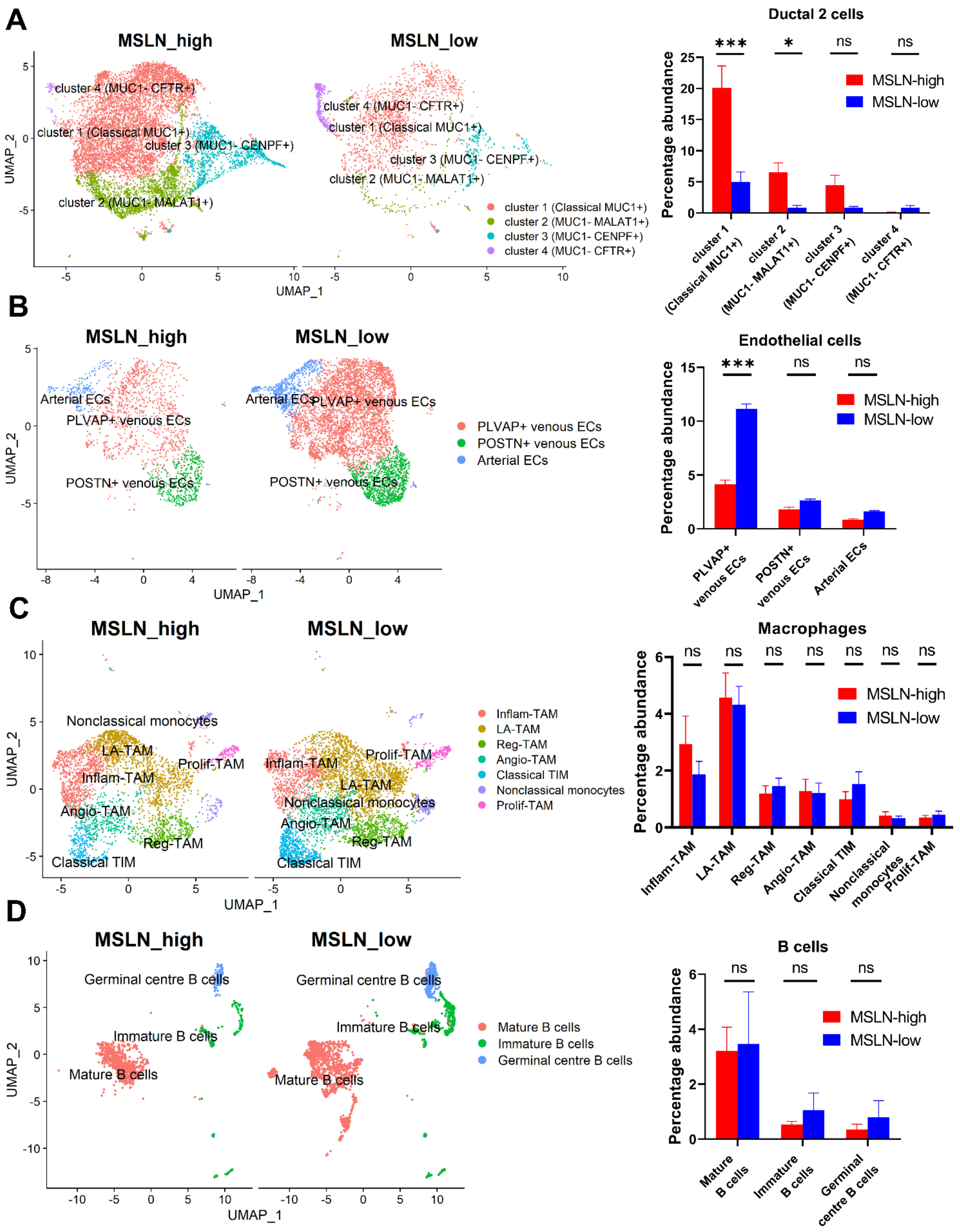
A
MSLN_high
MSLN_low
cluster 4 (MUC1- CFTR+)
cluster 1 (Classical MUC1+)
cluster 3 (MUC1- CENPF+)
cluster 2 (MUC1- MALAT1+)
UMAP_1
UMAP_2
Ductal 2 cells
Percentage abundance
MSLN-high
MSLN-low
***
*
ns
B
Arterial ECs
PLVAP+ venous ECs
POSTN+ venous ECs
Endothelial cells
C
Nonclassical monocytes
LA-TAM
Prolif-TAM
Inflam-TAM
Angio-TAM
Reg-TAM
Classical TIM
Macrophages
D
Germinal centre B cells
Immature B cells
Mature B cells
B cells

Supplementary Figure 6. Subtype analysis of other cell types in the single cell RNA-sequencing dataset. Cells were subset from the parental dataset, re-clustered, and subtypes were annotated manually. Data were visualized as UMAP comparison between MSLN-high and MSLN-low groups (left) and differences in subtype abundances were quantified (right). Percentage abundance (percentage out of total cells) of ductal 2 cell subtypes (**A**), endothelial cell subtypes (**B**), macrophage subtypes (**C**), and B cell subtypes (**D**) were shown. Mean ± SEM. Statistical testing by student's t-tests (*$p < 0.05$; ***$p < 0.001$; ns, not significant).

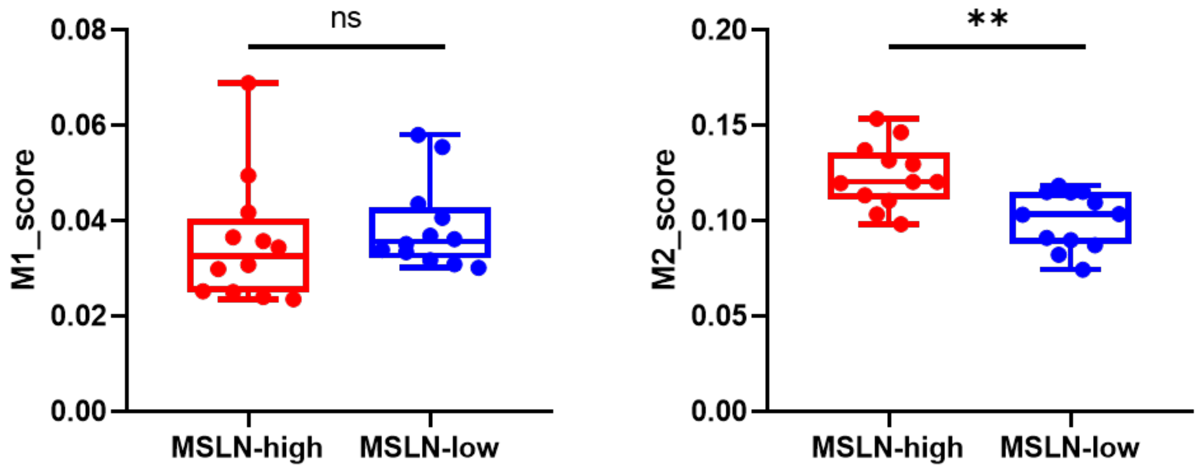

Supplementary Figure 7. M1 and M2 polarization scores of the macrophage population from the single cell RNA-sequencing dataset. Scores were evaluated for each sample via UCell using M1 (left) and M2 (right) gene signatures, respectively. Statistical testing by student's t-tests (**$p < 0.01$; ns, not significant).

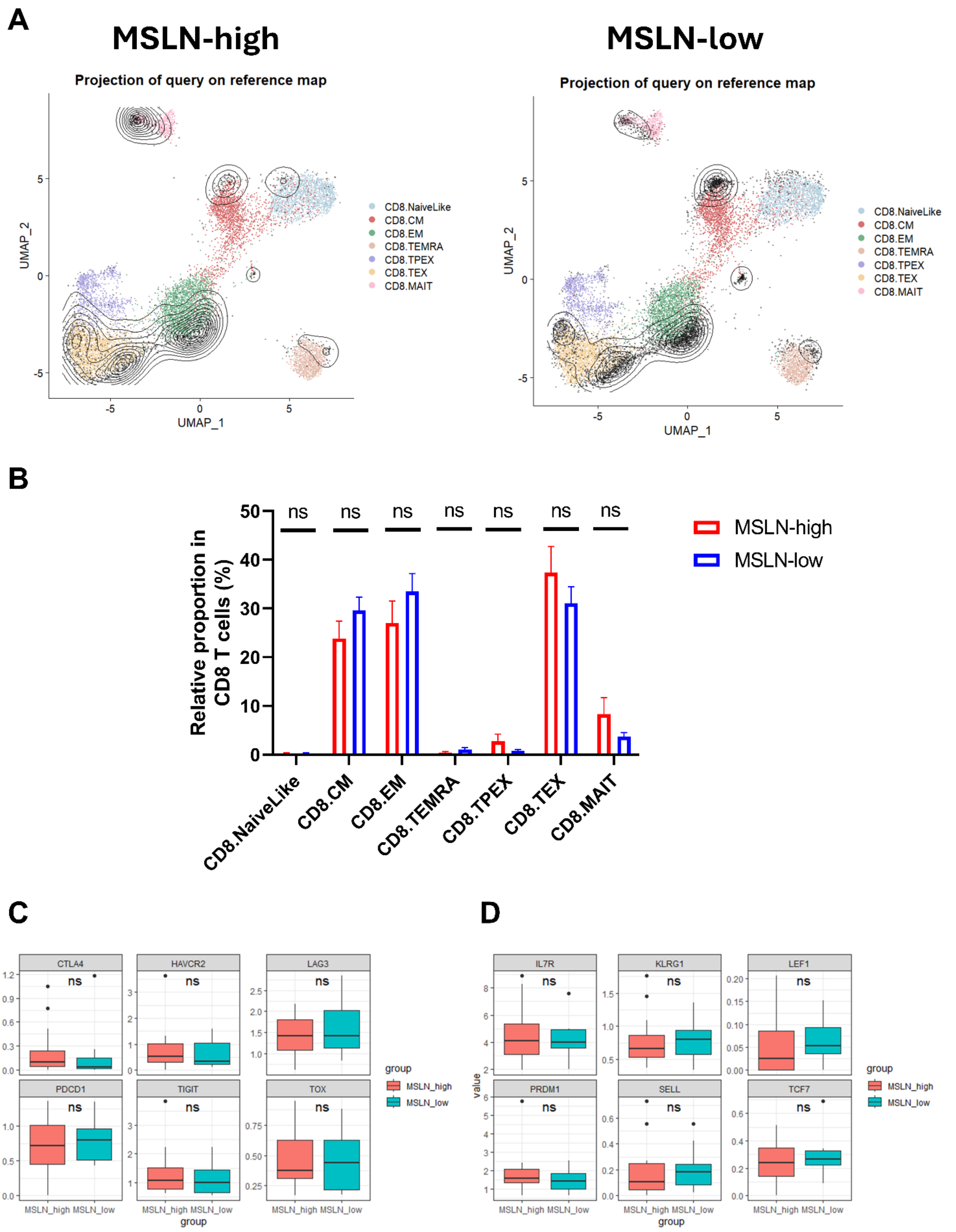

Supplementary Figure 8. Exhaustion and memory phenotypes of CD8 T cells from the single-cell RNA sequencing dataset. **(A)** UMAP comparison of CD8 T cells from MSLN-high (left) and MSLN-

low (right) groups, when projected onto a reference CD8 atlas. **(B)** Differences in the relative proportion of projected CD8 T cell subsets between the MSLN-high and MSLN-low groups. Expression of phenotypic markers for exhaustion **(C)** and memory **(D)** between MSLN-high and MSLN-low groups. Mean ± SEM. Statistical testing by student's t-tests (ns, not significant)

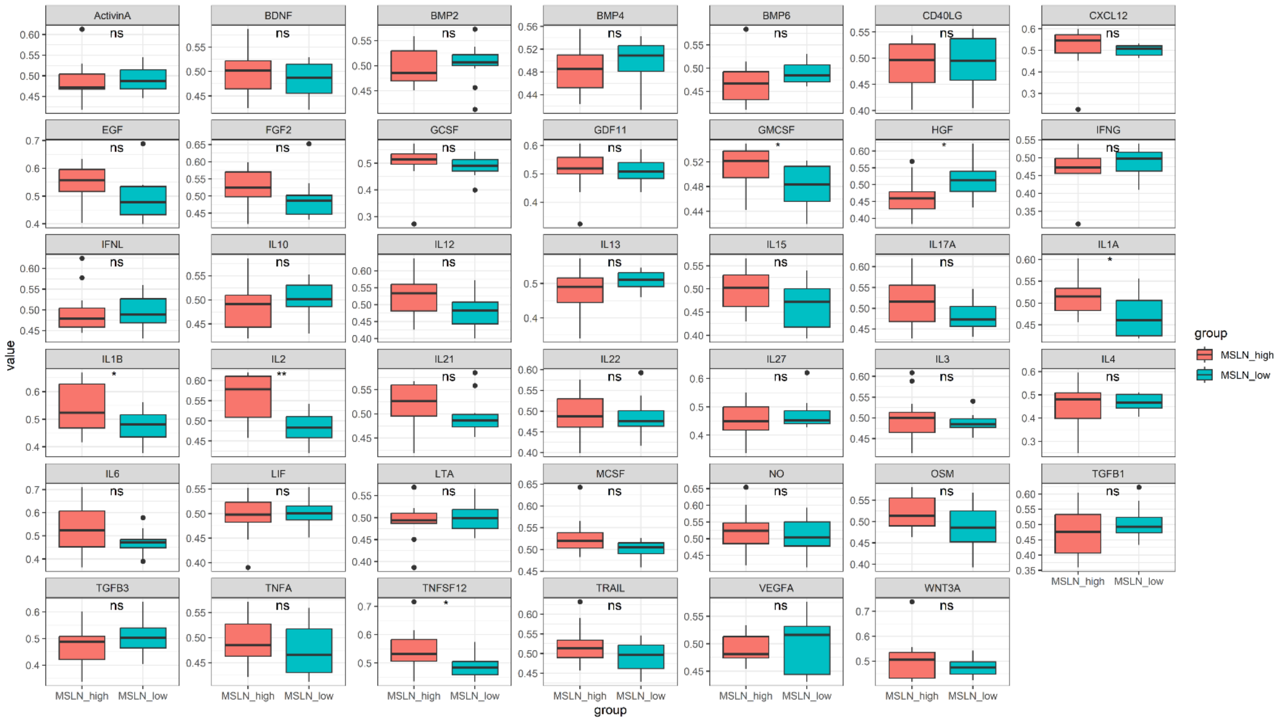

Supplementary Figure 9. Cytokine signalling profiles of CD8 T cells from the single-cell RNA sequencing dataset. Predicted activities of 41 cytokine signalling pathways were inferred for each sample using the CytoSig platform. Statistical testing by student's t-tests (**p* < 0.05; ns, not significant).

A

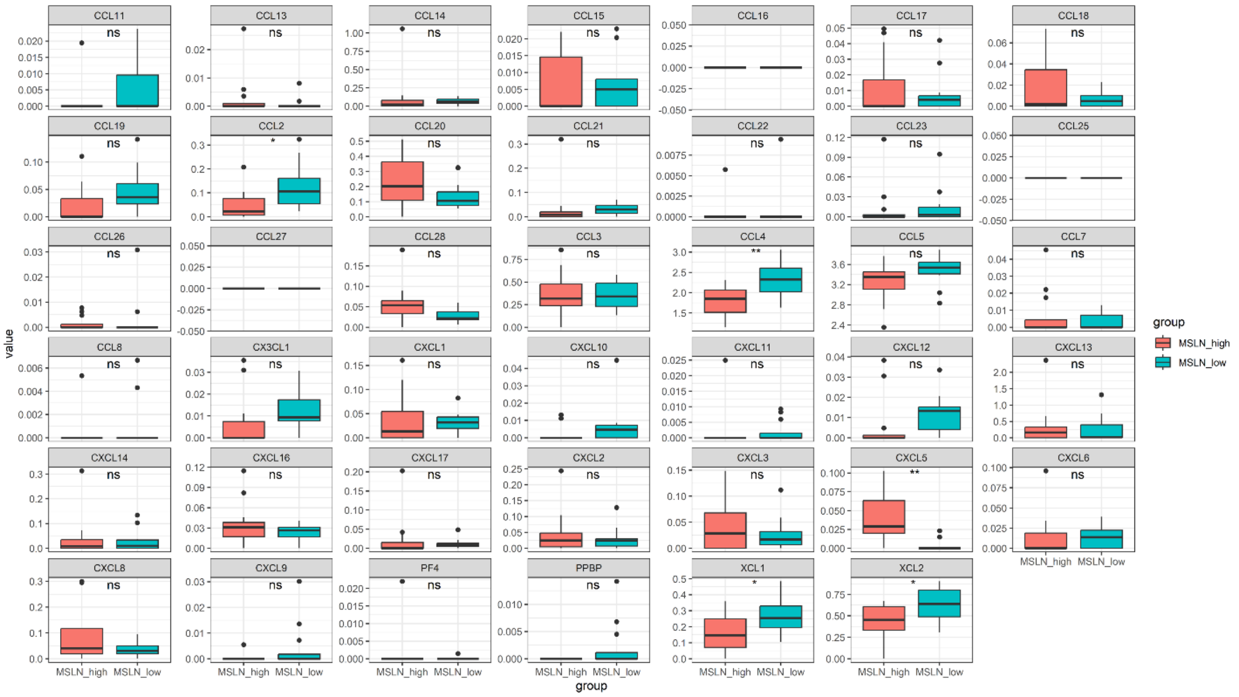

B

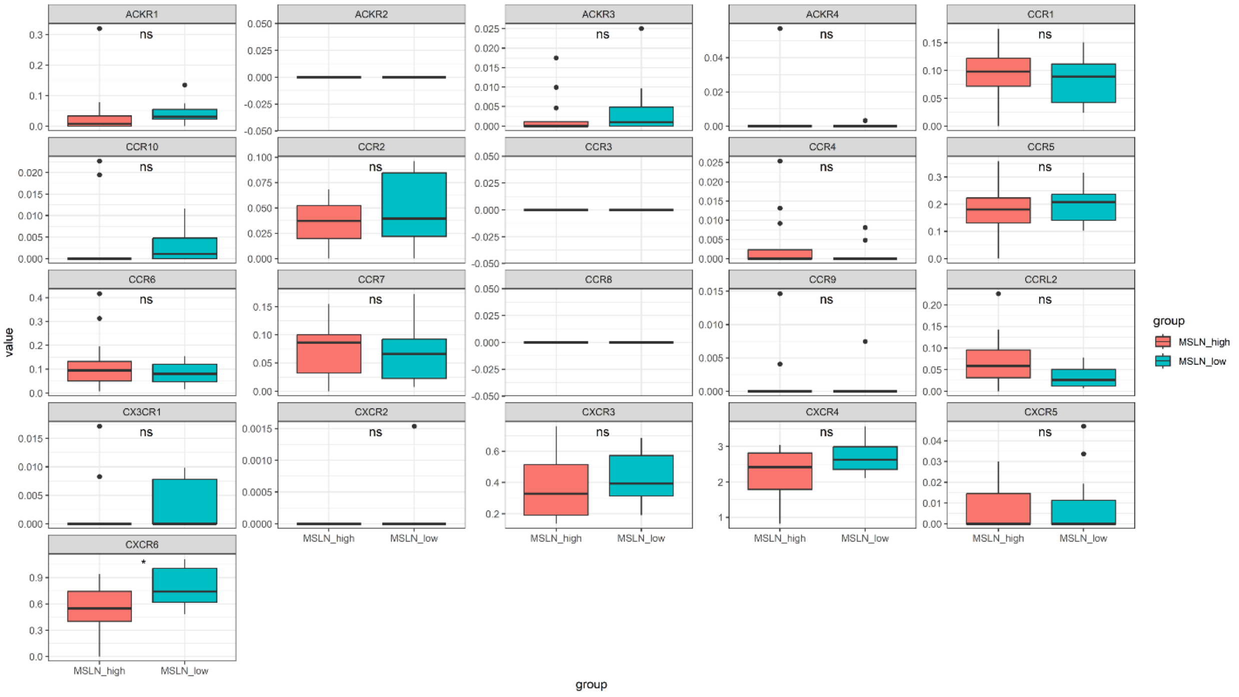

Supplementary Figure 10. Chemokine profiles of CD8 T cells from the single-cell RNA sequencing dataset. Expression of all human chemokines **(A)** and chemokine receptors **(B)** were examined for each sample. Differences were compared between samples in the MSLN-high and MSLN-low groups. Statistical testing by student's t-tests (**p* < 0.05; ***p* < 0.01; ns, not significant).

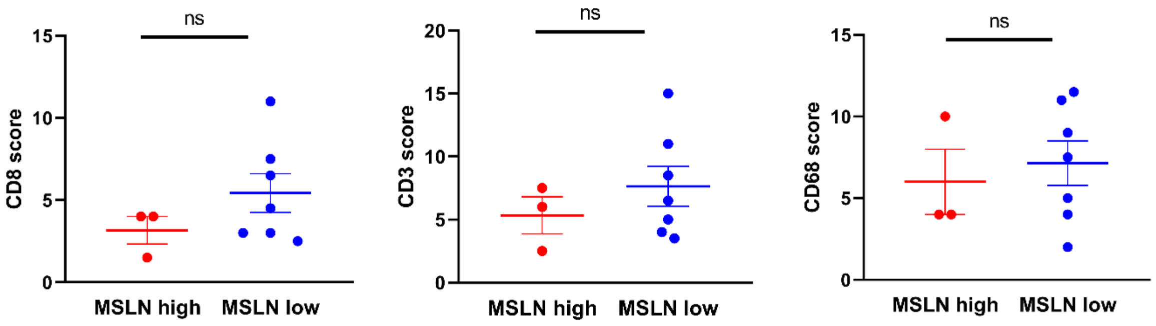

Supplementary Figure 11. Differences in CD8, CD3, and CD68 scores between samples with high and low mesothelin (MSLN) expression, stratified using a H-score threshold of 62. Expressions were assessed by immunohistochemistry on formalin-fixed, paraffin-embedded sections of surgical specimens, and scored independently by pathologists. Mean ± SEM. Statistical testing by student's t-tests (ns, not significant).

2.2 Supplementary Tables

Supplementary Table 1: Associations between *MSLN* expression and clinicopathological characteristics in the human RNA-seq dataset

Parameter		Total (*n*=126)	MSLN-high (*n*=63)	MSLN-low (*n*=63)	P-value
Cohort, *n* (%) 126 (100)	Australian	55 (43.7)	26 (41.3)	29 (46.0)	ns
	Canadian	71 (56.3)	37 (58.7)	34 (54.0)	
Age, *n* (%) 125 (99.2)	< 70	75 (60.0)	39 (61.9)	36 (58.1)	ns
	≥ 70	50 (40.0)	24 (38.1)	26 (41.9)	
Sex, *n* (%) 124 (98.4)	Male	68 (54.0)	39 (61.9)	29 (46.0)	ns
	Female	57 (45.2)	23 (36.5)	34 (54.0)	
Tumor stage, *n* (%) 66 (52.4)	I	37 (36.3)	25 (37.9)	12 (33.3)	ns
	II	56 (54.9)	34 (51.5)	22 (61.1)	
	III	5 (4.9)	5 (7.6)	0 (0.0)	
	IV	4 (3.9)	2 (3.0)	2 (5.6)	
Tumor location, *n* (%) 68 (54.0)	Head	51 (75.0)	23 (79.3)	28 (71.8)	ns
	Body	8 (11.8)	2 (6.9)	6 (15.4)	
	Tail	5 (7.4)	3 (10.3)	2 (5.1)	
	Duct*	1 (1.5)	0 (0)	1 (2.6)	
	Other parts	3 (4.4)	1 (3.4)	2 (5.1)	
Tumor differentiation, *n* (%) 123 (97.6)	Well differentiated	15 (12.2)	7 (11.1)	8 (13.3)	ns
	Moderately differentiated	68 (55.3)	33 (52.4)	35 (58.3)	
	Poorly differentiated	39 (31.7)	22 (34.9)	17 (28.3)	
	Undifferentiated*	1 (0.8)	1 (1.6)	0 (0)	
Treatment, *n* (%) 110 (87.3)	Surgery	28 (25.5)	14 (24.6)	14 (26.4)	ns
	Chemotherapy	35 (31.8)	21 (36.8)	14 (26.4)	
	Chemotherapy + radiation therapy	14 (12.7)	7 (12.3)	7 (13.2)	
	No treatment	33 (30.0)	15 (26.3)	18 (34.0)	
Response, *n* (%) 117 (92.9)	Complete remission/NED	22 (18.8)	10 (17.5)	12 (20.0)	ns
	Stable disease	17 (14.5)	10 (17.5)	7 (11.7)	
	Progressive disease/relapses	78 (66.7)	37 (64.9)	41 (68.3)	
Relapse type, *n* (%) 73 (57.9)	Local recurrence	14 (19.2)	9 (25.7)	5 (13.2)	ns
	Distant recurrence/metastasis	48 (65.8)	19 (54.3)	29 (76.3)	

	Local recurrence & distant metastasis	11 (15.1)	7 (20.0)	4 (10.5)

*Group was excluded from chi-squared analysis due to small number of samples (n = 1).

MSLN, mesothelin; PDAC, pancreatic ductal adenocarcinoma; NED, no evidence of disease; ns, not significant
